# Complete mitochondrial genomes of two gecko species, *Gekko hokouensis* and *Gekko japonicus* (Squamata, Gekkonidae)

**DOI:** 10.1080/23802359.2015.1137809

**Published:** 2016-06-20

**Authors:** Shuangli Hao, Danna Yu, Jun Ping, Huabin Zhou, Yongpu Zhang

**Affiliations:** a College of Life and Environmental Science, Wenzhou University, Wenzhou, China;; b Institute of Ecology, Zhejiang Normal University, Jinhua, China

**Keywords:** *Gekko japonicas*, *Gekko hokouensis*, Gekkonidae, mitogenome

## Abstract

The Gekkonidae is the second-most-numerous Family in Sauria and widely distributed around the world. In this paper, the complete mitochondrial genomes of two gecko species, *Gekko hokouensis* and *Gekko japonicas*, were sequenced. The lengths of the two mitochondrial genomes (*G. hokouensis* and *G. japonicus*) are 17 956 bp and 17 769 bp, respectively. These mitochondrial genomes both contain 13 protein-coding genes, two ribosomal RNA genes, 22 transfer RNA genes and a non-coding region (control region). The overall base compositions of the H-strand of *G. hokouensis* and *G. japonicus* are 32.1% A, 26.7% T, 27.0% C, 14.2% G and 31.6% A, 25.2% T, 28.6% C, 14.7% G, respectively. Phylogenetic analysis showed that *G. hokouensis* is a sister clade with *G. swinhonis*. *G. japonicus* has a close phylogenetic relationship to the clade of *G. hokouensis* and *G. swinhonis*.

Sixteen species of genus *Gekko* (Squamata: Gekkonidae) is distributed in China (Cai et al. 2015). Four complete mitochondrial genomes of *Gekko* are available in GenBank data (Zhou et al. 2006; Qin et al. 2011; Li et al. 2013; Hao et al. 2015). To analyze the mt genomes of other *Gekko* species, we sequenced the mt genomes of *G. hokouensis* and *G. japonicus* collected from Luxi Island (27°59′N, 121°10′) and Chashan (27°55′N, 120°42′), Wenzhou, Zhejiang, China, respectively. Voucher specimens were deposited in Wenzhou University. The accession numbers of *G. hokouensis* and *G. japonicus* are KT005801 and KT005800 in GenBank, respectively.

The lengths of complete mitochondrial genomes of *G. hokouensis* and *G. japonicus* were 17 769 bp and 17 956 bp, respectively. Both mitogenomes encode 37 genes including 13 respiratory protein-coding genes, two rRNA genes and 22 tRNA genes and the major non-coding region. The gene arrangement structure and transcribing directions were identical to other geckos. The canonical cloverleaf secondary structures predicted by tRNAscan-SE online server (http://lowelab.ucsc.edu/tRNAscan-SE/) could be found in all tRNA genes except *tRNA^Phe^*, *tRNA^Cys^* and one *tRNA^Ser^*. *tRNA^Ser^* lacked dihydrouridine (DHU) arm, which was a common feature observed in vertebrate animals (Meganathan et al. 2011).

Twelve protein-coding genes, 14 tRNAs and two rRNAs are transcribed from the heavy strand (H-strand), whereas *ND6* and the other eight tRNAs are located on the light strand (L-strand). The overall base compositions of the H-strand of *G. hokouensis* and *G. japonicus* are 32.1% A, 26.7% T, 27.0% C, 14.2% G and 31.6% A, 25.2% T, 28.6% C, 14.7% G, respectively. The overall A + T content of mt genomes are 58.8% for *G. hokouensis* and 56.8% for *G. japonicus*. The overall AT and GC skews for *G. hokouensis* and *G. japonicus* are 0.091, −0.312 and 0.112, −0.321, respectively. The 12S and 16S rRNA genes of the *G. hokouensis* and *G. japonicus* are located between *tRNA^Phe^* and *tRNA^Leu^* genes, and separated by *tRNA^Val^* gene. The control regions of *G. hokouensis* and *G. japonicus* located between the *tRNA^Phe^* and *tRNA^Pro^* genes are 2390 bp and 2539 bp in length with the A + T content of 63.0% and 64.6%, respectively.

To elucidate the phylogenetic relationships of gekkonids, 22 sequences of the complete (or nearly complete) mitochondrial genomes were obtained from GenBank database. Bayesian inference (BI) and maximum likelihood (ML) trees were analyzed by using MrBayes 3.1.2 (Huelsenbeck & Ronquist 2001) and PAUP* 4.0b10 (Swofford 2002) (Figure 1). *Coleonyx variegatus*, *Hemitheconyx caudicinctus* and *H. taylori* were selected as outgroups. Phylogenetic analysis showed that *G. hokouensis* was a sister clade with *G. swinhonis* and the clade of *G. hokouensis* and *G. swinhonis* was a sister with *G. japonicus*. The monophyly of *Gekko* is well supported, but the monophyly of Gekkonidae is failed for *Phyllodactylus unctus* (Phyllodactylidae) within Gekkonidae, which is inconformity to the results of Pyron et al. (2013). More species of Phyllodactylidae are need to further study the relationship of Gekkonidae and Phyllodactylidae.

**Figure 1.**
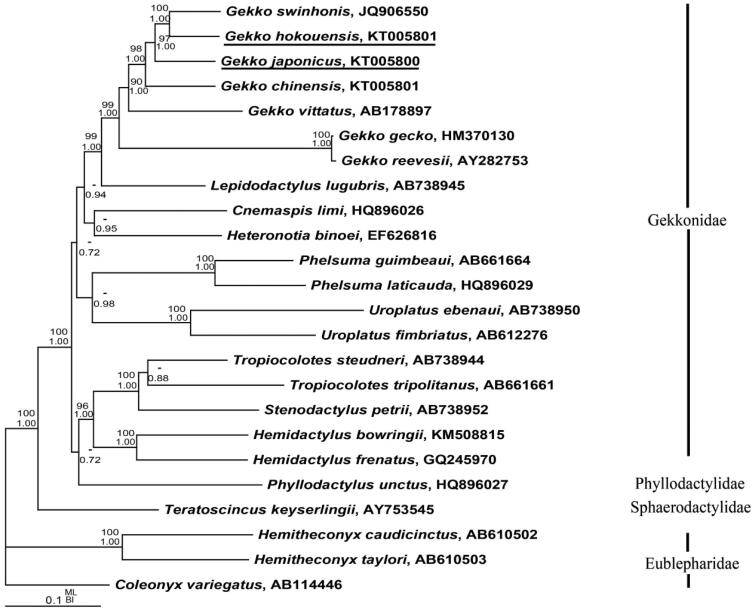
Bayesian inference (BI) and maximum likelihood (ML) phylogeny results of the 24 species from 13 mt protein-coding genes were analyzed. The nodal support above branches is shown as posterior probabilities from BI and bootstrap percentages from ML. Branch lengths and topology are from the Bayesian analysis.
